# Crystal structure of di­bromo­meth­oxy­seselin (DBMS), a photobiologically active pyran­ocoumarin

**DOI:** 10.1107/S2056989017006132

**Published:** 2017-04-28

**Authors:** A. K. Bauri, Sabine Foro, A. F. M. M. Rahman

**Affiliations:** a Bio-Organic Division, Bhabha Atomic Research Centre, Trombay, Mumbai 400 085, India; b Institute of Materials Science, Darmstadt University of Technology, Alarich-Weiss-Strasse 2, D-64287 Darmstadt, Germany; c Department of Applied Chemistry & Chemical Engineering, University of Dhaka, Dhaka-1000, Bangladesh

**Keywords:** crystal structure, di­bromo­meth­oxy­seseline (DBMS), seseline: bromination, bromo product, π–π stacking, C—H⋯O inter­actions

## Abstract

The title compound, C_15_H_14_Br_2_O_4_ [systematic name: *rac*-(9*S*,10*R*)-3,9-dibromo-10-methoxy-8,8-dimethyl-9,10-dihydropyrano[2,3-*h*]chromen-2(8*H*)-one], is a pyran­ocoumarin derivative formed by the bromination of seselin, which is a naturally occurring angular pyran­ocoumarin isolated from the Indian herb *Trachyspermum stictocarpum*. In the mol­ecule, the benzo­pyran ring system is essentially planar, with a maximum deviation of 0.044 (2) Å for the O atom. The di­hydro­pyran ring is in a half-chair conformation and the four essentially planar atoms of this ring form a dihedral angle of 4.6 (2)° with the benzo­pyran ring system. In the crystal, mol­ecules are linked by weak C—H⋯O hydrogen bonds, forming chains propagating along [010]. In addition, π–π stacking inter­actions, with centroid–centroid distances of 3.902 (2) and 3.908 (2) Å, link the hydrogen-bonded chains into layers parallel to (001).

## Abstract 2

The title compound, a bromo derivative of pyran­ocoumarin, possesses photobiological activity. It was formed by bromination of seselin by using NBS in MeOH at room temperature. In the crystal, mol­ecules are linked by π–π stacking inter­actions and weak C—H⋯O inter­actions, forming layers parallel to (001).

## Chemical context   

The title compound is a substituted product of seselin containing two bromine atoms and a meth­oxy group. This class of pyran­ocoumarins have an absorption band in the near-UV region due to the presence of extended conjugated double bonds and exhibit photomutagenic (Appendino *et al.*, 2004 ▸) and photocarcinogenic properties to bind with the purin base of DNA in a living cell to yield photoadducts (Conforti *et al.*, 2009 ▸). Based on the properties of these mol­ecules, they are employed for the treatment of numerous inflammatory skin diseases such as atopic dermatitis and the pigment disorders vitiligo and psoriasis on exposure to ultra violet (UV) radiation in photodynamic therapy (PDT). It has also been found that as a result of their strong ability for absorption of UV radiation, they are utilized as photoprotective agents to prevent the absorption of harmful UV radiation by the skin in the form of a variety of sun-screening lotions widely used in dermatological applications in the cosmetic and pharmaceutical industries (Chen *et al.*, 2007 ▸, 2009 ▸). In addition to these activities, anti­proliferative activity and photo-toxicity of related coumarin mol­ecules has been reported against numerous cancer cell lines such as HL60, A431 (Conconi *et al.*, 1998 ▸). Inhibited proliferation in the human hepatocellular carcinoma cell line has also been reported (March *et al.*, 1993 ▸). Recently, this type of mol­ecule has been connected as a spacer with porphyrin moieties to obtain a scaffold-type macromolecule (mol­ecular nanotweezers) and has been employed to study the inter­action (host–guest inter­action) with fullerenes such as C_60_ and C_70_ (Banerjee *et al.*, 2014 ▸; Ghosh *et al.*, 2014 ▸) in supra­molecular chemistry and material science. Mol­ecular tweezers containing a coumarin moiety showed better quantum yield and fluorescence absorption as a result of the presence of the extended conjugated enone of pyran­ocoumarin. As part of our ongoing studies in this area, we herein describe the synthesis and structure of the title mol­ecule.
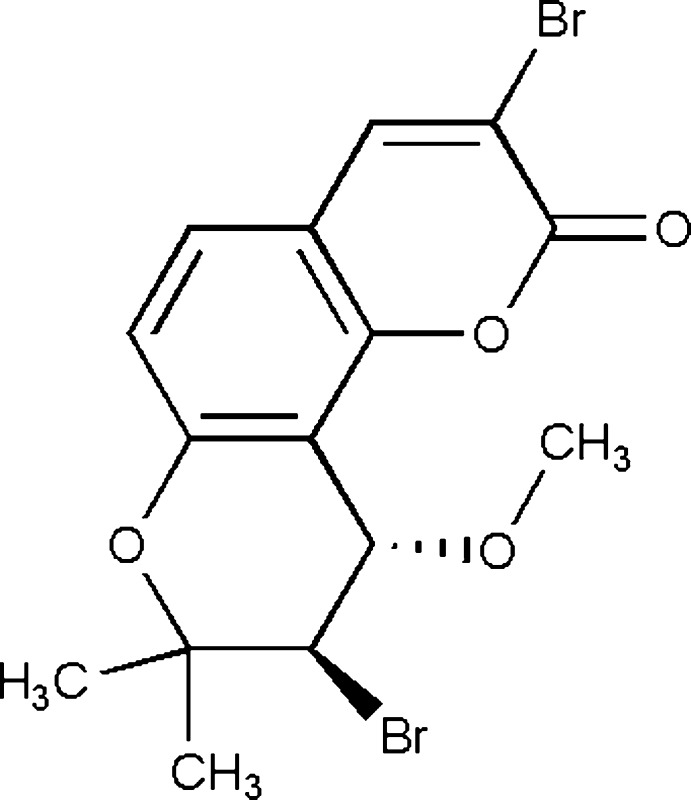


## Structural commentary   

**Figure 1:**
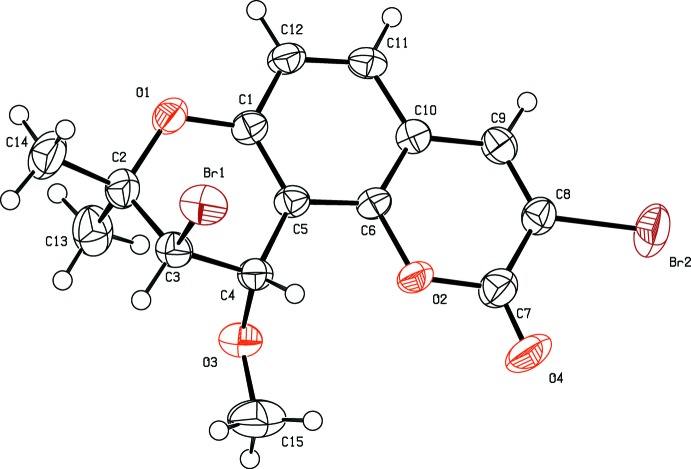
The mol­ecular structure of the title compound, showing the atom labelling and displacement ellipsoids drawn at the 50% probability level

The title mol­ecule (Fig. 1 ▸) is composed of three different types of rings *viz.* benzene, pyran and di­hydro­pyran. The benzo­pyran ring system C1/C5–C12/O2 is essentially planar with a maximum deviation of 0.044 (2) Å for atom O2. The di­hydro­pyran ring C1–C5/O1 is in a half-chair conformation and atoms C2 and C3 deviate by −0.385 (4) and 0.280 (4) Å from the plane through the other four essentially planar atoms (mean deviation 0.003 Å), which makes a dihedral angle of 4.6 (2)° with the benzo­pyran ring system. The relative stereochemistry at atoms C3 and C4 is *R*/*S* and *S*/*R*.

## Supra­molecular features   

**Figure 2:**
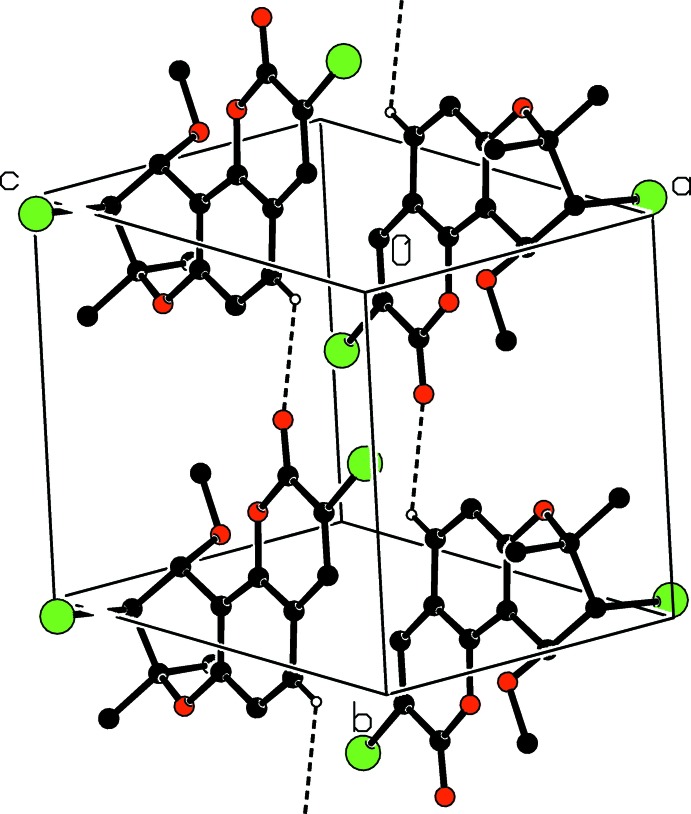
Part of the crystal structure with weak C—H⋯O hydrogen bonds shown as dashed lines. Only the H atoms involved in hydrogen bonds are shown.

**Figure 3:**
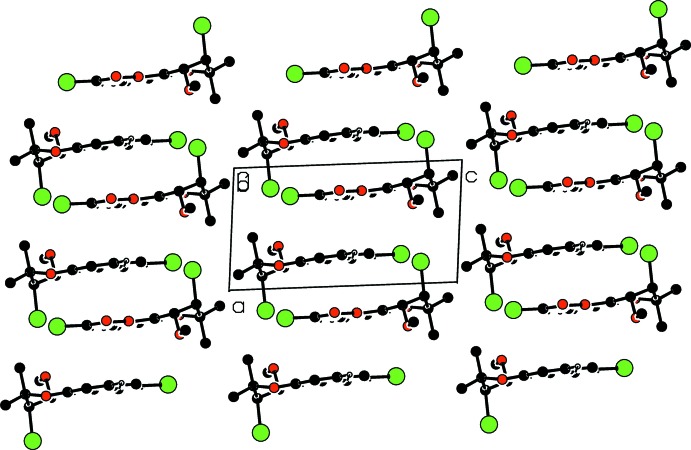
Part of the crystal structure showing layers of mol­ecules parallel to (001).

In the crystal, mol­ecules are linked by weak C—H⋯O hydrogen bonds (Table 1 ▸), forming chains propagating along [010] (Fig. 2 ▸). In addition, π–π stacking inter­actions with centroid–centroid distances *Cg*1⋯*Cg*1(2 − *x*, −*y*, 1 − *z*) of 3.902 (2) Å and *Cg*1⋯*Cg*2(1 − *x*, −*y*, 1 − *z*) of 3.908 (2) Å where *Cg*1 and *Cg*2 are the centroids of the C1/C5/C6/C10–C12 and O2/C6–C10 rings, respectively, link the hydrogen-bonded chains, forming layers parallel (001) (Fig. 3 ▸).

## Database survey   

A search of the Cambridge Structural Database (CSD, Version 5.38, update November, 2016; Groom *et al.*, 2016 ▸) gave more than thirty five hits for both linear and angular pyran­ocoumarin (psoralen class) structures. They include closely related structures [CSD refcodes AMYROL (Kato, 1970 ▸), FUGVOS (Thailambal & Pattabhi, 1987 ▸), AMYROL01 (Bauri *et al.*, 2006 ▸, 2017 ▸)] and a number of structures with various substituents at C3 and C4, many of which are natural products.

## Synthesis and crystallization   

The title compound is a colourless solid substance formed on bromination of the naturally occurring seseline isolated from the methanol extract of *T. stictocarpum* by means of column chromatography over SiO_2_ gel with gradient elution by using a mixture of the binary solvents hexane and ethyl acetate. The bromination was conducted using NBS in methanol at room temperature with continuous stirring by means of mechanical stirrer over a period of 12 h. The reaction product was worked up by the usual method to yield crude product, which was then purified by solvent elution to yield the title compound. A colourless prism-shaped crystal was obtained after recrystallization (×3) from ethyl acetate:hexane (1:4) at room temperature by slow evaporation of the solvents. NMR analysis: ^1^H NMR data (CDCl_3_, 200 MHz): δ_H_ 8.02 (*s*, 1H, H-9), 7.32 (*d*, 1H, *J* = 8.80 Hz, H-12), 6.82 (*d*, 1H, *J* = 8.80 Hz, H-11), 5.36 (*d*, 1H, *J* = 6.8 Hz, H-4), 4.26 (*d*, 1H, *J* = 6.8 Hz, H-3), 3.56 (*s*, 3H, –OCH_3_, H-13), 1.50 (*s*, 3H, CH_3_, H-13), 1.54 (*s*, 3H, CH_3_, H-14).

## Refinement   

Crystal data, data collection and structure refinement details are summarized in Table 2 ▸. H atoms were included in calculated positions and treated as riding atoms with C—H = 0.93–0.98 Å with U_iso_(H) = 1.2*U*_eq_(C).

## Supplementary Material

Crystal structure: contains datablock(s) I. DOI: 10.1107/S2056989017006132/lh5842sup1.cif

Structure factors: contains datablock(s) I. DOI: 10.1107/S2056989017006132/lh5842Isup2.hkl

Supporting information file. DOI: 10.1107/S2056989017006132/lh5842Isup3.cml

CCDC reference: 1545541

Additional supporting information:  crystallographic information; 3D view; checkCIF report

## Figures and Tables

**Table 1:** Hydrogen-bond geometry (Å, °)

*D*—H⋯*A*	*D*—H	H⋯*A*	*D*⋯*A*	*D*—H⋯*A*
C11—H11⋯O4^i^	0.93	2.57	3.188 (6)	124

Symmetry code: (i) 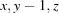.

**Table 2:** Experimental details

Crystal data	
Chemical formula	C_15_H_14_Br_2_O_4_
*M*_r_	418.08
Crystal system, space group	Triclinic, *P* 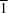
Temperature (K)	299
*a*, *b*, *c* (Å)	7.119 (1), 8.519 (1), 13.366 (2)
α, β, γ (°)	105.34 (2), 90.45 (1), 103.38 (2)
*V* (Å^3^)	758.4 (2)
*Z*	2
Radiation type	Mo *K*α
μ (mm^−1^)	5.36
Crystal size (mm)	0.20 × 0.20 × 0.16

Data collection	
Diffractometer	Oxford Diffraction Xcalibur single-crystal X-ray diffractometer with a Sapphire CCD detector
Absorption correction	Multi-scan (*CrysAlis RED*; Oxford Diffraction, 2009 ▸)
*T*_min_, *T*_max_	0.364, 0.423
No. of measured, independent and observed [*I* > 2σ(*I*)] reflections	5172, 2764, 2144
*R*_int_	0.015
(sin θ/λ)_max_ (Å^−1^)	0.602

Refinement	
*R*[*F* ^2^ > 2σ(*F* ^2^)], *wR*(*F* ^2^), *S*	0.035, 0.116, 0.85
No. of reflections	2764
No. of parameters	193
H-atom treatment	H-atom parameters constrained
Δρ_max_, Δρ_min_ (e Å^−3^)	0.46, −0.42

Computer programs: *CrysAlis CCD* and *CrysAlis RED* (Oxford Diffraction, 2009 ▸), *SHELXS97* and *SHELXL97* (Sheldrick, 2008 ▸) and *PLATON* (Spek, 2009 ▸).

## supplementary crystallographic information

### Crystal data

**Table:**

C_15_H_14_Br_2_O_4_	*Z* = 2
*M**_r_* = 418.08	*F*(000) = 412
Triclinic, *P*1	*D*_x_ = 1.831 Mg m^−^^3^
Hall symbol: -P 1	Mo *K*α radiation, λ = 0.71073 Å
*a* = 7.119 (1) Å	Cell parameters from 2165 reflections
*b* = 8.519 (1) Å	θ = 2.6–27.9°
*c* = 13.366 (2) Å	µ = 5.36 mm^−^^1^
α = 105.34 (2)°	*T* = 299 K
β = 90.45 (1)°	Prism, colourless
γ = 103.38 (2)°	0.20 × 0.20 × 0.16 mm
*V* = 758.4 (2) Å^3^	

### Data collection

**Table:**

Oxford Diffraction Xcalibur single-crystal X-ray diffractometer with a Sapphire CCD detector	2764 independent reflections
Radiation source: fine-focus sealed tube	2144 reflections with *I* > 2σ(*I*)
Graphite monochromator	*R*_int_ = 0.015
Rotation method data acquisition using ω and phi scans.	θ_max_ = 25.4°, θ_min_ = 2.6°
Absorption correction: multi-scan (CrysAlis RED; Oxford Diffraction, 2009)	*h* = −8→8
*T*_min_ = 0.364, *T*_max_ = 0.423	*k* = −8→10
5172 measured reflections	*l* = −16→12

### Refinement

**Table:**

Refinement on *F*^2^	Primary atom site location: structure-invariant direct methods
Least-squares matrix: full	Secondary atom site location: difference Fourier map
*R*[*F*^2^ > 2σ(*F*^2^)] = 0.035	Hydrogen site location: inferred from neighbouring sites
*wR*(*F*^2^) = 0.116	H-atom parameters constrained
*S* = 0.85	*w* = 1/[σ^2^(*F*_o_^2^) + (0.1*P*)^2^] where *P* = (*F*_o_^2^ + 2*F*_c_^2^)/3
2764 reflections	(Δ/σ)_max_ = 0.004
193 parameters	Δρ_max_ = 0.46 e Å^−^^3^
0 restraints	Δρ_min_ = −0.42 e Å^−^^3^

### Special details

**Table:**

Geometry. All esds (except the esd in the dihedral angle between two l.s. planes) are estimated using the full covariance matrix. The cell esds are taken into account individually in the estimation of esds in distances, angles and torsion angles; correlations between esds in cell parameters are only used when they are defined by crystal symmetry. An approximate (isotropic) treatment of cell esds is used for estimating esds involving l.s. planes.
Refinement. Refinement of F^2^ against ALL reflections. The weighted R-factor wR and goodness of fit S are based on F^2^, conventional R-factors R are based on F, with F set to zero for negative F^2^. The threshold expression of F^2^ &gt; 2sigma(F^2^) is used only for calculating R-factors(gt) etc. and is not relevant to the choice of reflections for refinement. R-factors based on F^2^ are statistically about twice as large as those based on F, and R- factors based on ALL data will be even larger.

### Fractional atomic coordinates and isotropic or equivalent isotropic displacement parameters (Å^2^)

**Table:**

	*x*	*y*	*z*	*U*_iso_*/*U*_eq_	
Br1	1.17651 (6)	0.02924 (6)	0.16407 (4)	0.04731 (18)	
Br2	0.74960 (8)	0.46658 (6)	0.74871 (4)	0.0602 (2)	
O1	0.8143 (4)	−0.2513 (3)	0.2144 (2)	0.0405 (7)	
O2	0.7746 (4)	0.2766 (3)	0.4357 (2)	0.0399 (7)	
O3	0.6525 (4)	0.1428 (3)	0.2086 (2)	0.0416 (7)	
O4	0.7816 (6)	0.5300 (4)	0.5337 (3)	0.0694 (11)	
C1	0.7877 (5)	−0.1487 (5)	0.3085 (3)	0.0322 (8)	
C2	0.7930 (6)	−0.1956 (5)	0.1231 (3)	0.0407 (9)	
C3	0.8945 (5)	−0.0109 (5)	0.1422 (3)	0.0345 (8)	
H3	0.8663	0.0240	0.0807	0.041*	
C4	0.8264 (5)	0.1028 (5)	0.2368 (3)	0.0319 (8)	
H4	0.9274	0.2070	0.2619	0.038*	
C5	0.7908 (5)	0.0188 (5)	0.3224 (3)	0.0305 (8)	
C6	0.7703 (5)	0.1103 (4)	0.4232 (3)	0.0299 (8)	
C7	0.7715 (6)	0.3884 (5)	0.5309 (3)	0.0437 (10)	
C8	0.7530 (6)	0.3147 (5)	0.6187 (3)	0.0357 (9)	
C9	0.7392 (5)	0.1528 (5)	0.6078 (3)	0.0352 (9)	
H9	0.7232	0.1108	0.6656	0.042*	
C10	0.7488 (5)	0.0428 (5)	0.5074 (3)	0.0305 (8)	
C11	0.7447 (5)	−0.1286 (5)	0.4888 (3)	0.0345 (9)	
H11	0.7290	−0.1781	0.5435	0.041*	
C12	0.7636 (6)	−0.2230 (5)	0.3909 (3)	0.0355 (9)	
H12	0.7605	−0.3362	0.3790	0.043*	
C13	0.5762 (7)	−0.2223 (6)	0.0942 (4)	0.0553 (12)	
H13A	0.5609	−0.1819	0.0348	0.066*	
H13B	0.5182	−0.1619	0.1518	0.066*	
H13C	0.5142	−0.3397	0.0780	0.066*	
C14	0.8775 (8)	−0.3121 (6)	0.0381 (4)	0.0590 (13)	
H14A	1.0133	−0.2949	0.0553	0.071*	
H14B	0.8597	−0.2880	−0.0270	0.071*	
H14C	0.8128	−0.4265	0.0325	0.071*	
C15	0.6827 (8)	0.2953 (7)	0.1849 (5)	0.0697 (16)	
H15A	0.7553	0.3827	0.2421	0.084*	
H15B	0.5602	0.3182	0.1723	0.084*	
H15C	0.7537	0.2904	0.1238	0.084*	

### Atomic displacement parameters (Å^2^)

**Table:**

	*U*^11^	*U*^22^	*U*^33^	*U*^12^	*U*^13^	*U*^23^
Br1	0.0397 (3)	0.0566 (3)	0.0543 (3)	0.0166 (2)	0.0127 (2)	0.0253 (2)
Br2	0.0801 (4)	0.0457 (3)	0.0433 (3)	0.0097 (2)	0.0166 (2)	−0.0031 (2)
O1	0.0586 (18)	0.0296 (14)	0.0347 (15)	0.0149 (13)	0.0110 (13)	0.0074 (12)
O2	0.0640 (19)	0.0262 (14)	0.0344 (15)	0.0166 (13)	0.0096 (13)	0.0114 (11)
O3	0.0400 (15)	0.0435 (17)	0.0481 (17)	0.0152 (13)	0.0043 (13)	0.0197 (14)
O4	0.127 (3)	0.0293 (18)	0.060 (2)	0.0305 (19)	0.021 (2)	0.0158 (15)
C1	0.0314 (19)	0.031 (2)	0.036 (2)	0.0078 (16)	0.0052 (16)	0.0104 (17)
C2	0.051 (2)	0.039 (2)	0.031 (2)	0.0126 (19)	0.0068 (18)	0.0062 (17)
C3	0.036 (2)	0.040 (2)	0.032 (2)	0.0129 (17)	0.0100 (16)	0.0139 (17)
C4	0.035 (2)	0.031 (2)	0.033 (2)	0.0101 (16)	0.0032 (16)	0.0112 (16)
C5	0.0319 (19)	0.0293 (19)	0.033 (2)	0.0083 (16)	0.0065 (15)	0.0120 (16)
C6	0.0313 (19)	0.0245 (19)	0.035 (2)	0.0061 (15)	0.0039 (16)	0.0096 (15)
C7	0.051 (3)	0.038 (3)	0.043 (2)	0.016 (2)	0.008 (2)	0.0089 (19)
C8	0.038 (2)	0.034 (2)	0.034 (2)	0.0105 (17)	0.0062 (17)	0.0061 (16)
C9	0.035 (2)	0.040 (2)	0.032 (2)	0.0077 (17)	0.0048 (16)	0.0122 (17)
C10	0.0287 (18)	0.032 (2)	0.032 (2)	0.0075 (16)	0.0067 (15)	0.0103 (16)
C11	0.036 (2)	0.033 (2)	0.039 (2)	0.0070 (17)	0.0029 (17)	0.0181 (17)
C12	0.042 (2)	0.027 (2)	0.041 (2)	0.0099 (17)	0.0039 (18)	0.0131 (17)
C13	0.055 (3)	0.050 (3)	0.048 (3)	−0.005 (2)	−0.001 (2)	0.008 (2)
C14	0.088 (4)	0.041 (3)	0.044 (3)	0.019 (2)	0.020 (3)	0.002 (2)
C15	0.068 (4)	0.065 (4)	0.091 (4)	0.029 (3)	−0.003 (3)	0.035 (3)

### Geometric parameters (Å, º)

**Table:**

Br1—C3	1.963 (4)	C6—C10	1.388 (5)
Br2—C8	1.876 (4)	C7—C8	1.463 (6)
O1—C1	1.371 (4)	C8—C9	1.328 (5)
O1—C2	1.440 (5)	C9—C10	1.432 (5)
O2—C6	1.375 (4)	C9—H9	0.9300
O2—C7	1.377 (5)	C10—C11	1.408 (5)
O3—C15	1.386 (5)	C11—C12	1.369 (5)
O3—C4	1.431 (4)	C11—H11	0.9300
O4—C7	1.183 (5)	C12—H12	0.9300
C1—C5	1.384 (5)	C13—H13A	0.9600
C1—C12	1.401 (5)	C13—H13B	0.9600
C2—C3	1.524 (6)	C13—H13C	0.9600
C2—C14	1.526 (5)	C14—H14A	0.9600
C2—C13	1.538 (6)	C14—H14B	0.9600
C3—C4	1.533 (5)	C14—H14C	0.9600
C3—H3	0.9800	C15—H15A	0.9600
C4—C5	1.496 (5)	C15—H15B	0.9600
C4—H4	0.9800	C15—H15C	0.9600
C5—C6	1.394 (5)		
			
C1—O1—C2	117.6 (3)	C9—C8—C7	122.9 (4)
C6—O2—C7	123.4 (3)	C9—C8—Br2	122.2 (3)
C15—O3—C4	114.0 (3)	C7—C8—Br2	114.9 (3)
O1—C1—C5	122.4 (3)	C8—C9—C10	120.1 (4)
O1—C1—C12	115.6 (3)	C8—C9—H9	119.9
C5—C1—C12	122.0 (3)	C10—C9—H9	119.9
O1—C2—C3	111.0 (3)	C6—C10—C11	117.6 (3)
O1—C2—C14	104.5 (3)	C6—C10—C9	118.1 (3)
C3—C2—C14	113.4 (3)	C11—C10—C9	124.3 (3)
O1—C2—C13	109.0 (3)	C12—C11—C10	120.4 (3)
C3—C2—C13	109.7 (3)	C12—C11—H11	119.8
C14—C2—C13	109.1 (4)	C10—C11—H11	119.8
C2—C3—C4	113.0 (3)	C11—C12—C1	119.9 (3)
C2—C3—Br1	112.1 (3)	C11—C12—H12	120.0
C4—C3—Br1	107.3 (3)	C1—C12—H12	120.0
C2—C3—H3	108.1	C2—C13—H13A	109.5
C4—C3—H3	108.1	C2—C13—H13B	109.5
Br1—C3—H3	108.1	H13A—C13—H13B	109.5
O3—C4—C5	109.4 (3)	C2—C13—H13C	109.5
O3—C4—C3	110.3 (3)	H13A—C13—H13C	109.5
C5—C4—C3	110.5 (3)	H13B—C13—H13C	109.5
O3—C4—H4	108.8	C2—C14—H14A	109.5
C5—C4—H4	108.8	C2—C14—H14B	109.5
C3—C4—H4	108.8	H14A—C14—H14B	109.5
C1—C5—C6	116.3 (3)	C2—C14—H14C	109.5
C1—C5—C4	122.9 (3)	H14A—C14—H14C	109.5
C6—C5—C4	120.7 (3)	H14B—C14—H14C	109.5
O2—C6—C10	120.6 (3)	O3—C15—H15A	109.5
O2—C6—C5	115.6 (3)	O3—C15—H15B	109.5
C10—C6—C5	123.8 (3)	H15A—C15—H15B	109.5
O4—C7—O2	118.2 (4)	O3—C15—H15C	109.5
O4—C7—C8	127.1 (4)	H15A—C15—H15C	109.5
O2—C7—C8	114.7 (3)	H15B—C15—H15C	109.5
			
C2—O1—C1—C5	−16.8 (6)	C7—O2—C6—C10	4.5 (6)
C2—O1—C1—C12	165.3 (3)	C7—O2—C6—C5	−174.8 (3)
C1—O1—C2—C3	44.2 (5)	C1—C5—C6—O2	179.7 (3)
C1—O1—C2—C14	166.8 (3)	C4—C5—C6—O2	3.4 (5)
C1—O1—C2—C13	−76.7 (4)	C1—C5—C6—C10	0.4 (6)
O1—C2—C3—C4	−55.3 (4)	C4—C5—C6—C10	−176.0 (3)
C14—C2—C3—C4	−172.6 (4)	C6—O2—C7—O4	177.8 (4)
C13—C2—C3—C4	65.1 (4)	C6—O2—C7—C8	−3.0 (6)
O1—C2—C3—Br1	66.0 (3)	O4—C7—C8—C9	178.8 (5)
C14—C2—C3—Br1	−51.3 (4)	O2—C7—C8—C9	−0.3 (6)
C13—C2—C3—Br1	−173.5 (3)	O4—C7—C8—Br2	−1.1 (7)
C15—O3—C4—C5	142.1 (4)	O2—C7—C8—Br2	179.8 (3)
C15—O3—C4—C3	−96.1 (4)	C7—C8—C9—C10	2.2 (6)
C2—C3—C4—O3	−83.3 (4)	Br2—C8—C9—C10	−178.0 (3)
Br1—C3—C4—O3	152.6 (2)	O2—C6—C10—C11	179.7 (3)
C2—C3—C4—C5	37.8 (4)	C5—C6—C10—C11	−1.0 (6)
Br1—C3—C4—C5	−86.2 (3)	O2—C6—C10—C9	−2.5 (5)
O1—C1—C5—C6	−177.2 (3)	C5—C6—C10—C9	176.8 (3)
C12—C1—C5—C6	0.5 (6)	C8—C9—C10—C6	−0.8 (6)
O1—C1—C5—C4	−1.0 (6)	C8—C9—C10—C11	176.9 (4)
C12—C1—C5—C4	176.8 (3)	C6—C10—C11—C12	0.7 (5)
O3—C4—C5—C1	111.1 (4)	C9—C10—C11—C12	−177.0 (4)
C3—C4—C5—C1	−10.6 (5)	C10—C11—C12—C1	0.1 (6)
O3—C4—C5—C6	−72.7 (4)	O1—C1—C12—C11	177.1 (3)
C3—C4—C5—C6	165.6 (3)	C5—C1—C12—C11	−0.8 (6)

### Hydrogen-bond geometry (Å, º)

**Table:**

*D*—H···*A*	*D*—H	H···*A*	*D*···*A*	*D*—H···*A*
C11—H11···O4^i^	0.93	2.57	3.188 (6)	124

Symmetry code: (i) *x*, *y*−1, *z*.
